# A Secure Trust Establishment Scheme for Wireless Sensor Networks

**DOI:** 10.3390/s140101877

**Published:** 2014-01-22

**Authors:** Farruh Ishmanov, Sung Won Kim, Seung Yeob Nam

**Affiliations:** Department of Information and Communication Engineering, Yeungnam University, 214-1 Dae-dong, Gyeongsan-si, Kyongsan 712-749, Gyeongsangbuk-do, Korea; E-Mails: farruh.uzb@gmail.com (F.I.); synam@ynu.ac.kr (S.Y.N.)

**Keywords:** trust establishment, wireless sensor networks, misbehavior detection

## Abstract

Trust establishment is an important tool to improve cooperation and enhance security in wireless sensor networks. The core of trust establishment is trust estimation. If a trust estimation method is not robust against attack and misbehavior, the trust values produced will be meaningless, and system performance will be degraded. We present a novel trust estimation method that is robust against on-off attacks and persistent malicious behavior. Moreover, in order to aggregate recommendations securely, we propose using a modified one-step M-estimator scheme. The novelty of the proposed scheme arises from combining past misbehavior with current status in a comprehensive way. Specifically, we introduce an aggregated misbehavior component in trust estimation, which assists in detecting an on-off attack and persistent malicious behavior. In order to determine the current status of the node, we employ previous trust values and current measured misbehavior components. These components are combined to obtain a robust trust value. Theoretical analyses and evaluation results show that our scheme performs better than other trust schemes in terms of detecting an on-off attack and persistent misbehavior.

## 1. Introduction

The power of wireless sensor networks (WSNs) relies on distributed collaboration among sensor nodes for various tasks, such as event monitoring, relaying data, *etc*. [1,2]. Hence, it is important to maintain successful collaboration in order to maintain network functionality. Successful collaboration is assured only when all nodes operate in a trustworthy manner [3–5]. Trust establishment allows detection of trustworthy and untrustworthy nodes by evaluating them based on their behavior/performance. As sensor nodes often lack tamper-resistant hardware and are easily compromised, cryptographic solutions cannot ensure full protection of the network. Hence, trust establishment improves security by continuously monitoring node behavior/performance, evaluating the trustworthiness of the nodes and finding trustworthy nodes to collaborate with. Specifically, establishing trust in the network provides many benefits, such as the following [6]:

- Trust provides a solution for granting corresponding access control based on the quality of the sensor nodes and their services, which cannot be solved through traditional security mechanisms.
- Trust assists routing by providing reliable routing paths that do not contain malicious, selfish, or faulty nodes.
- Trust makes traditional security more robust and reliable by ensuring that only trustworthy nodes participate in authentication, authorization, or key management.

Recently, many trust establishment schemes have been proposed in various fields such as e-commerce, web-based services, peer-to-peer networks and WSNs. Basically, in WSNs trust is estimated periodically based on the number of instances of good and bad behavior counted during a certain time interval and using a certain method [3–8]. In addition, the number of instances of good and bad behavior during the previous time interval is added, but with a forgetting factor [3–8]. The problem with this kind of trust estimation method is that it focuses more on recent behavior of the node rather than comprehensively combining the nodes' past behavior with current behavior. As a consequence, a malicious node can easily remove any bad history by either displaying good behavior or waiting during subsequent time periods to increase its trust value, and in this way, continue attacking. For example, in an on-off attack, the malicious node alternates its behavior from good to bad and from bad to good so it is not detected while attacking. Moreover, persistence of the misbehavior is not considered under traditional trust estimation methods because trust values are obtained based on current instantaneous behavior, which does not indicate continuity of misbehavior. Specifically, only weight of measured misbehavior is considered rather than frequency of the misbehavior along with weight of measured misbehavior. For example, when measured misbehavior falls below a trust threshold, it can be detected at once; otherwise, it is not detected at all. Hence, when measured misbehavior is insignificant but persistent, it is not detected by traditional trust estimation methods. Detection of such misbehavior is important in WSNs, since a large number of nodes will misbehave due to faults in software and hardware [8]. Because nodes are error prone, they may get stuck malfunctioning for a long time [8]. Moreover, as sensor nodes often lack tamper-resistant hardware and are easily compromised, they may launch intelligent attacks against a trust-establishment mechanism. For example, a malicious node might misbehave for a long time, keeping its trust value above a trust threshold without being detected.

To overcome the aforementioned problems, we propose a novel trust estimation method that considers previous trust value, aggregated misbehavior and current measured misbehavior components to estimate the trust value of each node. The aggregated misbehavior component is a summation of periodically measured misbehavior, but with a forgetting factor. It helps to detect persistent misbehavior and an on-off attack, since it indicates the misbehavior history of the node comprehensively. So, if a node misbehaves continuously, then aggregated misbehavior will increase continuously over time till it reaches its maximum value,1, and its trust value will be decreased until it is under the trust threshold. If there is no misbehavior by a node in the current trust estimation time period, then aggregated misbehavior will be decreased, but with a forgetting factor, and the current trust value will be increased accordingly. However, the forgetting factor will be lower for aggregated misbehavior, if the node's trust value falls below the trust threshold. This is to mitigate the effect of an on-off attack and to punish malicious nodes. Moreover, current measured misbehavior and previous trust value emphasize recent behavior of the node. These three components are utilized to produce a robust trust value. To the best of our knowledge, this is the first persistent malicious detection trust establishment scheme. Moreover, we propose using a modified one-step M-estimator to securely aggregate recommendations. It is a lightweight scheme, yet robust against a bad-mouthing attack, which detects dishonest recommendations and excludes them before recommendation aggregation.

We prove the correctness and efficiency of our proposed method through theoretical analyses and evaluations. Evaluation results show that our proposed method can detect all kind of persistent malicious nodes provided the persistent measured misbehavior is equal or greater than 0.2. Moreover, under a given scenario, the proposed scheme can detect an on-off attack up to 70% of the time. For secure recommendation aggregation, the one-step M-estimator shows resilience against dishonest recommendations when they constitute up to 40% of the total number of recommendations. Hence, nodes can securely aggregate recommendations when dishonest recommendations account for up to 40% of the total recommendations.

The remainder of this paper is organized as follows: in Section 2, we present an overview of related work. Section 3 describes the proposed trust establishment scheme. Evaluation results and theoretical analyses of the proposed scheme are provided in Section 4 and Section 5. Section 6 concludes the paper.

## 2. Related Work

Recently, many trust establishment schemes have been proposed in various fields, such as e-commerce, web-based services, peer-to-peer networks and WSNs, which demonstrates the importance of trust establishment in general [9–14].

One of the earliest comprehensive trust establishment schemes which is called Group-Based Trust Management Scheme for Clustered Wireless Sensor Networks (GTMS) was proposed by Shaikh *et al*. [6]. The scheme works in three phases:

- Trust calculation at the node level
- Trust calculation at the cluster head (CH) level
- Trust calculation at the base station (BS) level

Nodes estimate trust value based on direct and indirect observations. A timing window mechanism is used to eliminate the effect of the time on trust values and to countermeasure on-off attacks. The timing window Δ*t*, which has several units, counts the number of successful and unsuccessful interactions. Using information in the time window, the trust value of node *y* at node *x* is estimated as follows [6]:

(1) $$T_{x,y}=[100\times (\frac{{\left(S_{x,y}\right)}^{2}}{\left(S_{x,y}+U_{x,y})(S_{x,y}+1\right)})]$$

where [·] is the nearest integer function, *S_x,y_* is the total number of successful interactions of node *x* with node *y* during time Δ*t*, and *U_x,y_* is the total number of unsuccessful interactions of node *x* with node *y* during time Δ*t*. After estimation of the trust value, a node will quantize trust into three states in the proposed mechanism: trusted, uncertain, and untrusted.

Each CH will periodically broadcast a request packet within its cluster to estimate global trust for its members. Upon receiving trust states from member nodes on their neighbor nodes, the CH will maintain these states in matrix form. After determining relative differences in the trust states of the node, a global value is assigned by the CH. The relative difference is emulated through a standard normal distribution.

The BS also maintains a record of past interactions with CHs, and the BS estimates trust for the CHs. The advantages of this scheme are that it is lightweight and energy-aware, which meets the requirements of WSNs. Furthermore, the authors proved that GTMS is resilient against cheating, bad behavior, and group attacks under the assumption that the number of unsuccessful interactions is equal to, or more than, the number of successful interactions. However, this may not always be true, because an attacking node usually tries as much as possible to avoid detection.

One of the more recent trust establishment schemes, ReTrust, is proposed by He *et al.* [15]. Similar to work by Shaikh *et al*. [6], the proposal also works in a two-tier architecture. The entire network is divided into cells, and each cell has member nodes and one manager node. In a certain cell, node *x* estimates a trust value for node *y* as follows [15]:

(2) $$T_{x,y}=[\alpha \times \frac{\sum_{j=1}^{m}\beta_{j}(1-p^{j})\times p^{j}}{\sum_{j=1}^{m}\beta_{j}(1-p^{j})})]$$

where *α* value determines the range and format of the trust value as [0, α] [15] and *m* is the number of units in a window-based forgetting mechanism. The authors use the window mechanism to forget previous actions. Moreover, they introduce an aging-factor parameter, *β_j_* , which is different for each time unit *m* in the window. *β_j_* is defined as *β_j_* = *φ^m^*^−^*^j^*, where *0*<*φ*<*1*. *p^j^* shows a successful interaction rate. It is estimated as follows [15]:

(3) $$p^{j}=\frac{s^{j}+1}{s^{j}+y^{j}+2}$$

where *s^j^* is the number of successful interactions during the *j*'th unit of the window, and *y^j^* is the number of unsuccessful interactions during the *j*'th unit of the window.

Velloso *et al*. [16] proposed another trust establishment approach that they called maturity-based trust management for mobile *ad hoc* networks. The main contribution of the paper was introducing the concept of relationship maturity, which improves the quality of a trust evaluation in the presence of mobility. According to the employed concept, recommendations by long-term neighbors are given more importance than recommendations by short-term neighbors. The trust level for node *b* given by node *a* is estimated as follows [16]:

(4) $$T_{a}(b)=(1-\alpha )Q_{a}(b)+\alpha R_{a}(b)$$

where *Q_a_*(*b*) is an observation-based derived trust value of node *a* about node *b*, from the range [0,1], and *R_a_*(*b*) represents aggregated value of the recommendations from all other neighbors. The variable *α* is a parameter that provides a relevant weight to each factor. (*b*) is given by:

$$Q_{a}(b)=\beta E_{a}(b)+(1-\beta )T_{a}(b)$$

where *E_a_* is the currently obtained trust value, and *T*_a_ represents the last trust value. The variable *β*, from the range [0,1], is a parameter that provides a relevant weight to each factor.

Moreover, the authors propose a recommendation exchange protocol to efficiently manage recommendation exchanges. It consists of three messages: a Trust Request (TREQ) message, a Trust Reply (TREP) message, and a Trust Advertisement (TA) message. TREQ is used to request recommendations from neighbors on a target node. Neighbors of the target node reply with a Trust Reply (TREP) message after waiting a random period of time, *tREP*, to avoid collisions and to wait for other TREQs. TA is used to inform its neighbors about a drastic change in trust value of a certain node during a trust update.

Even though this work has advantages, such as improving trust estimation in a mobile environment, the proposed scheme does not include a mechanism against on-off and bad-mouthing attacks. Since these attacks have a direct influence on estimated trust values, not considering the influence leads to incorrect decisions.

Feng *et al*. [17] proposed a node behavioral belief theory evaluation algorithm, which combines the method of node behavioral strategies and modified evidence theory. Based on the behaviors of sensor nodes and a variety of trust factors and coefficients related to the network application, both direct and indirect trust values are obtained by calculating a weighted average of trust factors. Specifically, the following factors are considered to estimate trust:

- Received packet rate
- Successfully sent packet rate
- Packet forwarding rate
- Data consistency
- Time relativity of context content in period *t*
- Node availability
- Security grade

Indirect trust is estimated by simply multiplying the trust value of the recommendation provider by the provided trust value. To integrate direct and indirect trust, Dempster–Shafer evidence theory is used. After obtaining trust values, fuzzy classification of trust values is performed as follows: first, trust is one of three states: completely distrust, uncertain, and completely trust. Second, according to the three states, it marks up three fuzzy subsets *T*1, *T*2 and *T*3 on the universe of nodes' trust value T ([0, 1]). The corresponding membership functions are *u*1(*t*)*, u*2(*t*) and *u*3(*t*), *u*1(*t*) + *u*2(*t*) + *u*3(*t*) = 1.

## 3. Secure Trust Establishment Scheme

### 3.1. Assumptions

We assume that nodes can monitor other nodes' activities within their communication range. For example, a node can overhear its neighbors' transmissions, and in this way, can detect whether the node is forwarding or dropping the packets. Moreover, trust value is estimated for each certain time interval by each node based on the results of monitoring within the trust estimation time interval. A malicious node acts intelligently, that is, it tries to keep its trust value in the trusted zone while attacking the network.

### 3.2. Observation-Based Trust Calculation

Trust is calculated based on either past interactions or past recommendations. A past interaction–based trust estimation method considers three factors to estimate the current trust value: current measured misbehavior, aggregated misbehavior, and previous trust value. Current measured misbehavior shows a node's behavior during the current time, whereas aggregated misbehavior and previous trust value demonstrate how much a node has misbehaved in the past. Current misbehavior of node *x* at time *t* by node *y* is measured as follows:

(6) $$a_{xy}(t)=\frac{b_{xy}}{b_{xy}+c_{xy}}$$

where *b_xy_* and *c_xy_* are the number of instances of bad behavior and the number of instances of good behavior of node *y* with node *x* within the Δ time interval.

Aggregated misbehavior, aggregates measured misbehavior over time using proposed method. It shows the persistency of the misbehavior. So, according to our proposed method if measured misbehavior is persistent, that is, it is always greater than predefined threshold, then each time aggregated misbehavior will be increased until it reaches to maximum value(that is one).Aggregated misbehavior of node *x* is estimated at time *t* by node *y* as follows:

(7) $$M_{xy}(t)=\{\begin{array}{l} \min\left\{\left[a_{xy}(t)+(1-S)\times M_{xy}(t-\Delta )\right],1\right\},\;\text{if}T_{xy}(t-\Delta )\geq Q,\\ \min\left\{\left[a_{xy}(t)+S\times M_{xy}(t-\Delta )\right],1\right\},\;\text{otherwise}, \end{array}$$

where *S* is forgetting factor for aggregated misbehavior, which ranges from [0.5, 1], 0.5 ≤ *S*≤ *1*. Our goal to define the forgetting factor in this way is to provide adaptability and improve the attack detection. For example, if a network designer wants to assign the same value for forgetting factor regardless of trust value, then he can assign 0.5. On the other hand, if he prefers to assign different values according to trust value, then the equation allows him to use this way, too. Hence, it provides a room for adaptability according to preference of the designer. According to Equation (7) once the node's trust value is under the trust threshold, aging factors for previous aggregated misbehavior will be different. It means the malicious node or on-off attack node requires a longer time to recover its trust value once it has been determined to be a malicious node. In order to estimate trust value, we use aggregated misbehavior, previous trust value, and current measured misbehavior to. While aggregated misbehavior focuses on the past misbehavior of the node, previous trust value and current measured misbehavior emphasize on the current status of the node. Then, node *y* estimates the trust value of node *x* at time *t* as follows:

(8) $$T_{xy}(t)=\{\begin{array}{ll} \frac{T_{xy}(t-\Delta )+\left(1-M_{xy}(t)\right)}{2+M_{xy}(t)}, & \text{if}\;a_{xy}(t)=0,\\ \frac{T_{xy}(t-\Delta )+\left(1-a_{xy}(t)\right)}{2+M_{xy}(t)}, & \text{otherwise}, \end{array}$$

where *T_xy_ (t-*Δ*)* is the trust value of node *x* by node *y* at time *t*-Δ. If current measured misbehavior is zero by node *y* on node *x* (that is, if there is no misbehavior currently by node *x*) then its previously aggregated misbehavior is used to estimate its current trust value. This is to protect the trust mechanism from an on-off attack and attacks similar to an on-off attack. Moreover, unlike a traditional trust estimation mechanism, our trust mechanism maintains previous trust value to estimate current trust value, which helps to track a node's behavior more accurately. After calculating the trust value, node *y* determine node's *x s*tatus based on its trust value as follows:

(9) $$Q(T_{xy}(t))=\left\{\begin{array}{ll} 1-f\leq T_{xy}(t)\leq 1 & \text{highly trusted}\\ 1-g\leq T_{xy}(t)<1-f & \text{trusted}\\ 1-h\leq T_{xy}(t)<1-g & \text{untrusted}\\ 0\leq T_{xy}(t)<1-h & \text{highly untrusted} \end{array}\right\}$$

where *f* < *g* < *h* < 1 and *f*, *g*, *h* can be tuned according to the system and security requirements to determine the node's state. Since these values depend on network and security conditions, it will be set accordingly. For instance, whether a node's trust value should be considered within the untrusted zone depends on the performance degradation tolerance of the network. Moreover, these parameters can be adaptive or fixed, depending on the security conditions. For instance, if the number of nodes with a trust value just above the trust threshold increases, degradation will be greater than in a situation where most of the nodes' trust values are in the highly trusted zone.

### 3.3. Recommendation-Based Trust Calculation

Nodes might need recommendations for certain nodes from other nodes for the following reasons:

- Lack of knowledge about the node, either due to a mobile environment or due to less interaction among the nodes.
- To combine recommendations with direct trust to obtain a comprehensive trust value.

If node *y* needs a recommendation about node *x*, it will ask only trustworthy nodes in unicast mode because it is more energy efficient than broadcast mode [18]. After receiving recommendations, it will aggregate all recommendations according to the defined method. Li *et al*. [18] showed that lightweight averaging algorithms perform better than complex aggregation algorithms. However, even though simple averaging performs better, in the presence of dishonest recommendations, an aggregated value can be distorted. Considering these factors, we use a modified one-step M-estimator (MOSE) [19,20], which is one of the robust measures of central tendency, to aggregate recommendations. It checks outliers using the median absolute deviation (MAD)-median rule, eliminates any found outliers, and then averages the remaining values [19]. MAD is measure of dispersion, or spread, around the median. In other words, it indicates the variability or diversity of the data around the median. It is more resilient to outliers in a data set than the standard deviation [19,20]. In order to determine the MAD for given dataset *X_1_*, *X_2_*,…, *X_i_*, absolute deviations from the median for each data is determined:

(10) $$\left|X_{i}-Md(X)\right|$$

where *X_i_* is *i^th^* data and *Md(X)* is median of the given data. Then, the MAD is defined as the median of the absolute deviations from the data's median:

(11) $$\text{MAD}=\text{media}n_{i}\left(\left|X_{i}-\text{media}n_{j}(X_{j})\right|\right)$$

Next, the median of these absolute values (median absolute deviation, or MAD) is estimated and scaled by a constant [19]:

(12) $$\text{MADN}=\frac{\text{MAD}}{0.675}$$

The recommendation is defined as an outlier or a dishonest recommendation if it is different from the majority of the group and the following statement is true [19]:

(13) $$\frac{\left|X_{i}-MD(x)\right|}{\text{MADN}}>K$$

where *Md*(*x*) is the median of the recommendation values, and *X_i_* is *i*th recommendation value. *K* is the threshold to determine the outlier and commonly used threshold value is 2.24. Any other threshold value can be used, which represents a stricter or a more tolerant criterion for determining an outlier. Moreover, we add one condition for a recommendation to be considered an outlier, or dishonest. The reason is that the outlier detection algorithm might determine that some recommendations are outliers even though they are not likely to be outliers. For example, the majority of the nodes might assess a certain node as trustworthy. However, when their recommendation values are highly dispersed, the outlier detection algorithm might determine some recommendations to be outliers since some values are far from the other values. Hence, considering majority opinion, we suggest not excluding from aggregation recommendations that belong to the majority. Moreover, for determining dishonest recommendations, simple averaging is performed on the remaining recommendations:

(14) $$R_{A}=\frac{\sum_{i=1}^{n}X_{i}}{n}$$

where *n* is number of recommendations, and *x_i_* is the *i*th recommendation.

## 4. Performance Evaluation

In this section, we evaluate and compare our proposed trust mechanism with other schemes proposed earlier. Evaluations are done for detection of persistent malicious behavior, on-off attacks, and bad-mouthing attacks. If an estimated trust value is under the trust threshold in persistent misbehavior or an on-off attack, we consider that misbehavior or attack have been detected. We compare our scheme with GTMS [6] and Retrust [15]. The former is one of the earliest comprehensive trust schemes for WSNs. On the other hand, the latter is one of the most recent comprehensive trust schemes.

### 4.1. Persistent Malicious Behavior Detection

Our scheme has a feature whereby it continuously decreases the trust value of a malfunctioning or malicious node when it persistently misbehaves. Misbehavior of the node is measured based on the proportion of the number of instances of bad behavior to the total number of behavior instances, $a=\frac{b}{b+c}$, where *b* the number of instances of bad behavior and *c* is the number of instances of good behavior. When measured misbehavior exceeds a predefined threshold value, *a* > *S*, the node is considered to be malicious under the trust estimation scheme. Sometimes a node might have a hardware or software problem that causes it to malfunction consistently [8]. For example, a node might drop a percentage of packets all the time, or it might always report false sensor data [8]. In this case, if the measured misbehavior exceeds the threshold, the malfunctioning node can be detected by traditional trust mechanisms; otherwise, it is considered a benevolent node even though it misbehaves persistently. Moreover, a malicious node might launch insignificant attacks consistently but keep its trust value above the trust threshold so it cannot be detected. When the attack is significant, it is easy to detect because it will be obvious from its performance within a short time. However, when the attack or misbehavior is insignificant but consistent, it is difficult to detect; it is even not possible for current trust estimation schemes because they do not consider continuity of the misbehavior in the trust estimation. Hence, detection of a consistent attack is important. To emulate consistent malicious behavior and to demonstrate detection of it, the parameters in Table 1 are used.

For each trust estimation time period, measured misbehavior is generated in random or fixed manner and trust is estimated based on generated misbehavior. We compare our trust estimation mechanism with GTMS [6] and Retrust [15]. Values of the system parameters such as trust threshold, forgetting factor, and time window are selected based on heuristic and previously defined values in the literature. For example, trust threshold is selected as about half of the maximum trust value in the literature [6,7,10,21–24]. Hence, in these references, defined trust threshold is between 0.4 and 0.8. In [21] the authors suggest that the most intuitive trust threshold is 0.5 when the maximum trust value is 1. Optimal trust threshold according to defined scenario in [24] is 0.6. The choice of value for forgetting factor remains largely heuristic and depends on the strategy of trust establishment [21]. Since forgetting factor is used mainly to combat on-off attack, authors use different values and different mechanisms to derive the value of forgetting factor according to their trust estimation and considerations [5,6,10,23]. Following the guidelines and suggestions in [5], we intuitively use forgetting factor as 0.6. Size of the time-window for GTMS and ReTrust is chosen to be 3 for the sake of simplicity.

**Figure 1.**
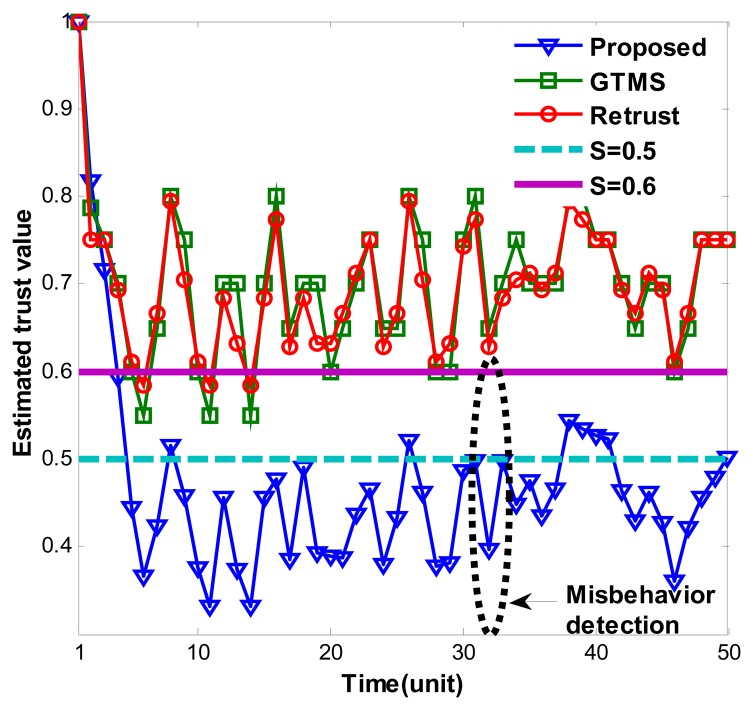
Persistent malicious behavior detection under random misbehavior.

Figure 1 shows estimated trust values over time under persistent malicious behavior. For each trust estimation period, measured misbehavior randomly measured between 0.1 and 0.4. As Figure 1 shows, our trust estimation mechanism decreases trust value gradually and keeps it under trust threshold when node shows consistent misbehavior. Trust values fluctuate because of the measured misbehavior. Since measured misbehavior is randomly generated between 0.1 and 0.4, that is, sometimes it can be high or low randomly, trust values fluctuate accordingly. Dynamicity of the trust values shows that our trust scheme considers efficiently current status of the node.

**Figure 2.**
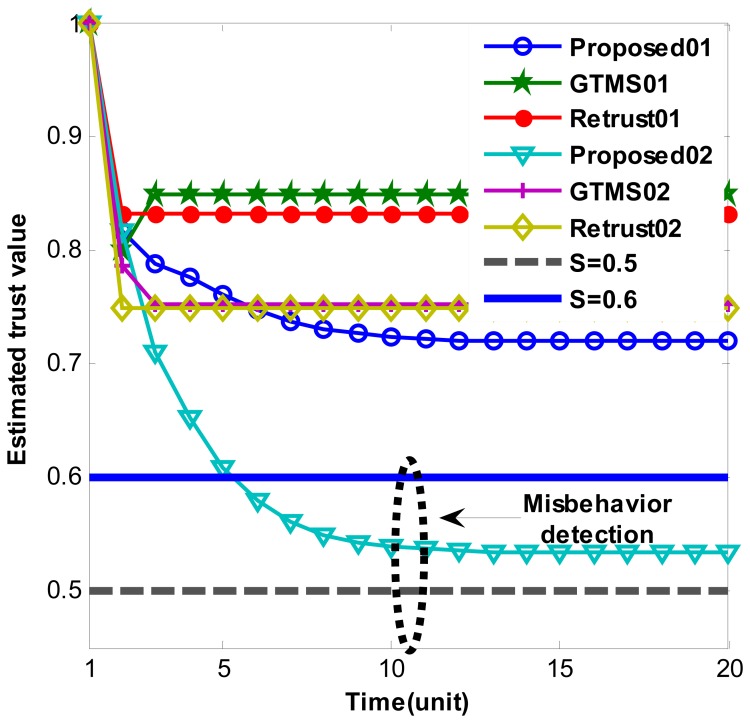
Persistent malicious behavior detection under constant misbehavior.

**Figure 3.**
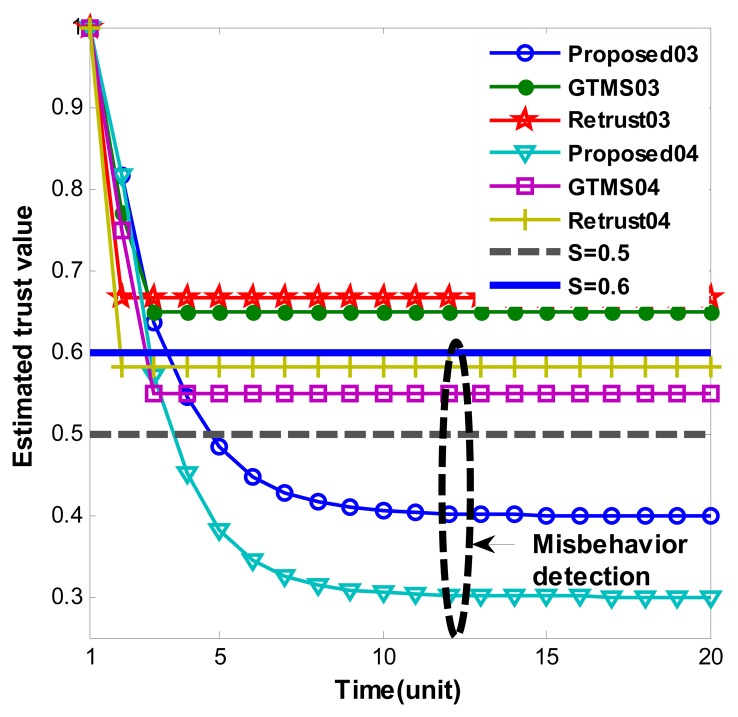
Persistent malicious behavior detection under constant misbehavior.

Figures 2 and 3 show persistent malicious behavior detection under different fixed measured misbehavior. Thus, measured misbehavior in each trust estimation period is fixed from 0.1 to 0.4. For instance, in Figure 2 ‘Proposed01’ means that performance of proposed scheme under fixed measured misbehavior such as 0.1. Hence, Figure 2 shows misbehavior detection when measured misbehavior fixed to 0.1 and 0.2. On the other hand, Figure 3 shows misbehavior detection when measured misbehavior fixed to 0.3 and 0.4, that is, measured misbehavior is set higher in Figure 3 evaluations. Important note from Figures 2 and 3 is that produced trust values in other schemes are constant even though misbehavior is persistent. On the other hand, our scheme gradually decreases trust value over time. When measured misbehavior fixed is to 0.1 in Figure 2, our scheme cannot detect such persistent misbehavior because estimated trust values do not go under trust threshold. The reason is that we intentionally design in this way to provide system tolerance. Otherwise, the scheme can be easily adapted to required parameters. In all other cases, our scheme can detect persistent malicious behavior as Figures 2 and 3 demonstrates. Trust values gradually go below trust threshold. Selected trust thresholds in the evaluations are default values because trust threshold is set to equal or greater than 0.5 normally in [6,7,9,14].

### 4.2. On-Off Attack Resilience Evaluation

**Figure 4.**
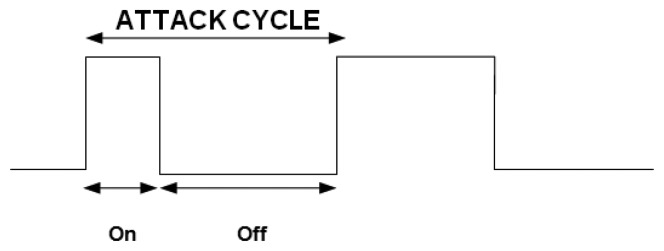
On-off attack cycle.

In this section, we evaluate the resilience of our trust model against on-off attacks. In an on-off attack, a malicious node alternates its behavior from malicious to normal and from normal to malicious so it remains undetected while causing damage. Thus, the attack cycle consists of two periods: on and off. An attack cycle is defined as “on” immediately followed by an “off” [25] (see Figure 4). When the attack is on, the malicious node launches attacks, and during the off period, either stops doing anything or only performs well. Since the on period has an implication on the trust value of the malicious node, it will try to increase its trust value during the off period by waiting or performing only good actions. Durations of both the on period and the off period can differ or be of equal length, depending on the malicious node's strategy.

The length of one attack cycle can be defined as follows:

$$L_{c}=A_{on}+A_{\text{off}}$$

where *L_c_* is the length of one attack cycle in terms of the time unit, and *A_on_* and *A_off_* are the lengths of the on period and off period in terms of the time unit, respectively.

To emulate behavior of an on-off attack node and evaluate the proposed trust scheme under an on-off attack, we use the parameters in Table 2. To make the emulation more realistic and fair, the duration of the on and off periods were generated randomly (that is, between one and five time units). Moreover, during the on period, the number of good and bad behaviors were randomly generated between ranges [5; 10] and [1; 5], respectively. Hence, in the worst case, the number of good and bad behaviors will be equal, otherwise the instances of good behavior always number more than bad behavior. The reason is that we assume that a malicious node tries to balance its misbehavior so it is not detected, and it can recover its trust value faster to attack again. Trust value is estimated after each time unit, and if an estimated trust value falls below the trust threshold, the node is considered untrustworthy for that period. To find the average detection rate of the attack, the sum of the number of times it was deemed untrustworthy is divided by the total experiment time.

**Figure 5.**
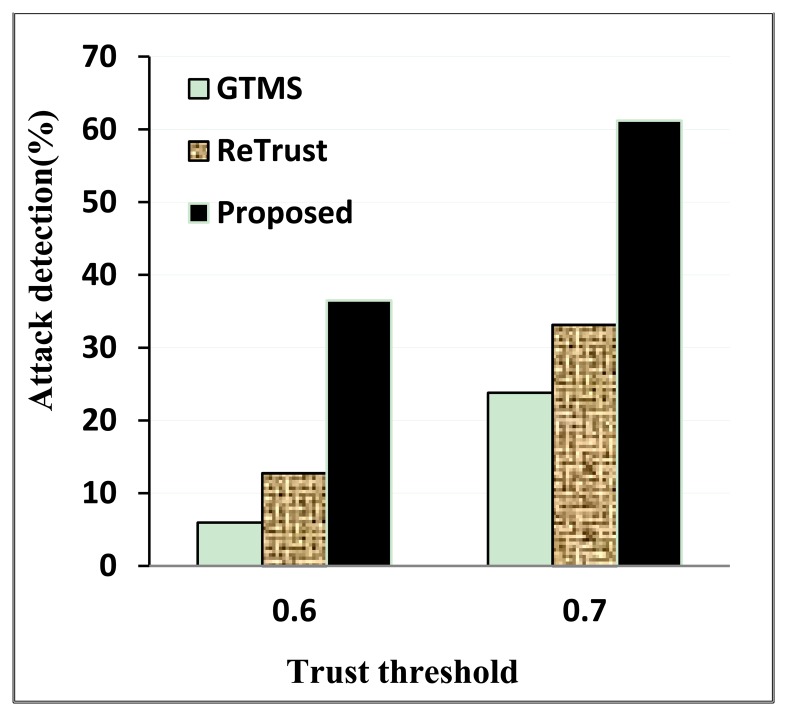
On-off attack detection.

As Figure 5 shows, the detection rate is the highest in our proposed scheme under both trust threshold scenarios. Since our proposed scheme decreases the trust value of the malicious node continuously, the recovery rate in the off period is slower when the trust value is under the trust threshold.

When the trust threshold is high, the on-off attack detection rate is also high. However, nodes might be rated as untrustworthy even though they might not actually be malicious nodes. That is why choosing a trust threshold requires considering all factors. Moreover, it is important to choose a trust recovery rate intelligently so that an on-off attack node has less chance to increase its trust value after the on period.

### 4.3. Bad-Mouthing Attack Resiliency

In a bad-mouthing attack, the malicious node provides a dishonest recommendation to decrease or increase the trust value of legitimate or malicious nodes, respectively. Moreover, the most dangerous scenario of such an attack is when a group of malicious nodes provide dishonest recommendations in a synchronized way (that is, the group of malicious nodes cooperate with each other in providing recommendations to decrease/increase trust values of certain legitimate/malicious nodes). Hence in this section, we evaluate resilience of our trust model against such bad-mouthing attacks. To emulate the bad-mouthing attack and detection of it, we use the following parameters (see Table 3):

Each time 10 recommendations are generated, the percentage of dishonest recommendations is set between 10% and 60%. We assume that the provided recommendations are for benevolent nodes. Hence, honest recommendation values are normally above the trust threshold. That is why we consider honest recommendation values as being between 0.6 and 0.9. Moreover, we assume that malicious nodes try to avoid being detected while providing dishonest recommendations. Hence, malicious nodes provide recommendations for benevolent nodes that are under the trust threshold, intending to distort the aggregated trust value (that is, to make it fall below the trust threshold). However, they act intelligently (that is, provided the recommendations will not be very low). Otherwise, detection of these dishonest recommendations will be obvious. Hence, we specifically chose the range for dishonest recommendations as [0.3; 05].

After generating honest and dishonest recommendations, outlier detection and aggregation is performed. In order to improve outlier detection, all recommendations are classified into two groups —trustworthy and untrustworthy—depending on the value of the recommendation. Moreover, one of the groups is determined the majority according to the number of recommendations in the group. Then, detected outliers are also classified into two groups—trustworthy and untrustworthy. If one of the groups belongs to the majority, then it is excluded from the outliers. The reason is that the outlier detection algorithm might determine some recommendations are outliers, even though they are not likely to be outliers. For example, a majority of the nodes might assess a certain node as trustworthy. However, when their recommendation values are highly dispersed, the outlier detection algorithm might determine some recommendations to be outliers because some values are far from the other values. Hence, considering majority opinion, we suggest not excluding recommendations that belong to the majority group. To find the outlier detection rate, the average outlier detection rate is estimated each time outlier detection is performed; then, a summation of the average is estimated. Among the criteria for recommendations to be aggregated correctly, the aggregated value should be above the trust threshold in the presence of dishonest recommendations. To demonstrate the outlier detection rate, we evaluate our proposed recommendation aggregation with different outlier thresholds and with different percentages of dishonest recommendations.

**Figure 6.**
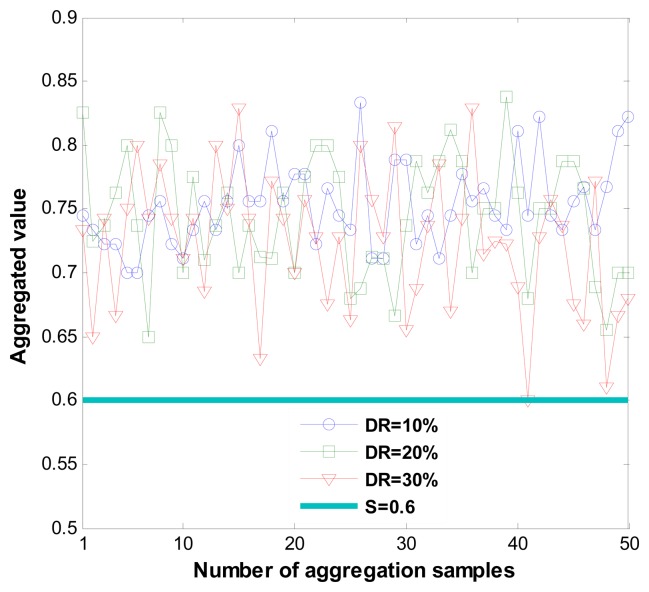
Recommendation aggregation in the presence of dishonest recommendations.

**Figure 7.**
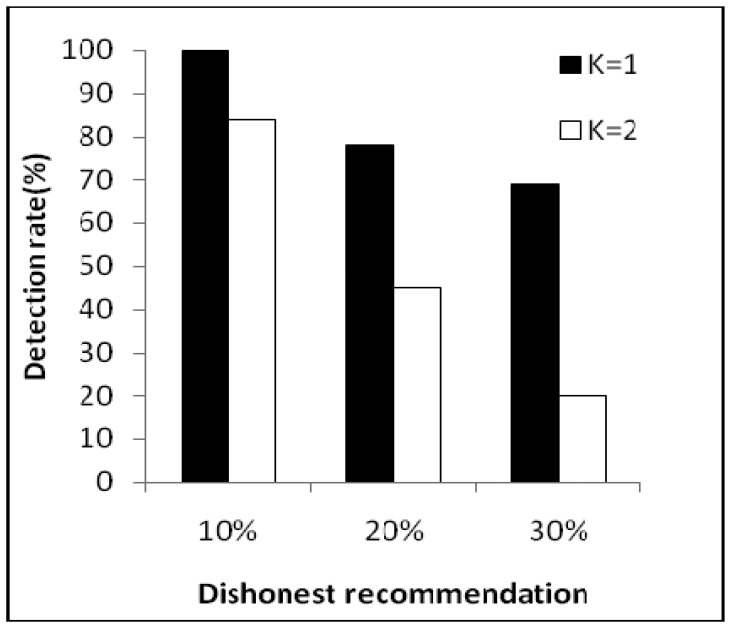
Dishonest recommendation detection.

Figure 6 shows correct recommendation aggregation in the presence of dishonest recommendations of between 10% and 30%. As we can see, with dishonest recommendations at up to 30%, the aggregated value is not distorted (that is, it is not under the trust threshold). Moreover, Figure 7 shows dishonest recommendation detection with different outlier detection in the presence of different percentages of dishonest recommendations. As the figure shows, when the threshold equals one (K = 1), the detection rate is more than 70% in the worst case.

**Figure 8.**
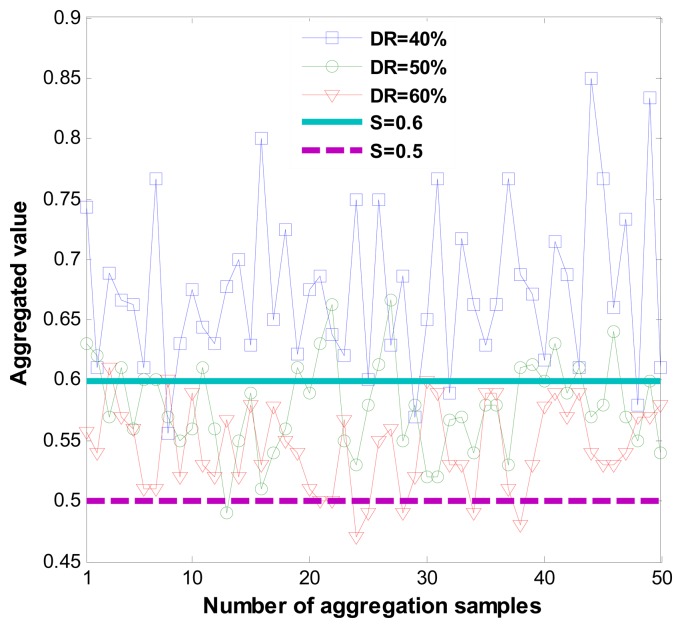
Recommendation aggregation in the presence of dishonest recommendations.

Figure 8 shows correct recommendation aggregation in the presence of dishonest recommendations that vary from 40% to 60%. Figure 8 demonstrates that when dishonest recommendations total 40%, aggregated values are not distorted.

**Figure 9.**
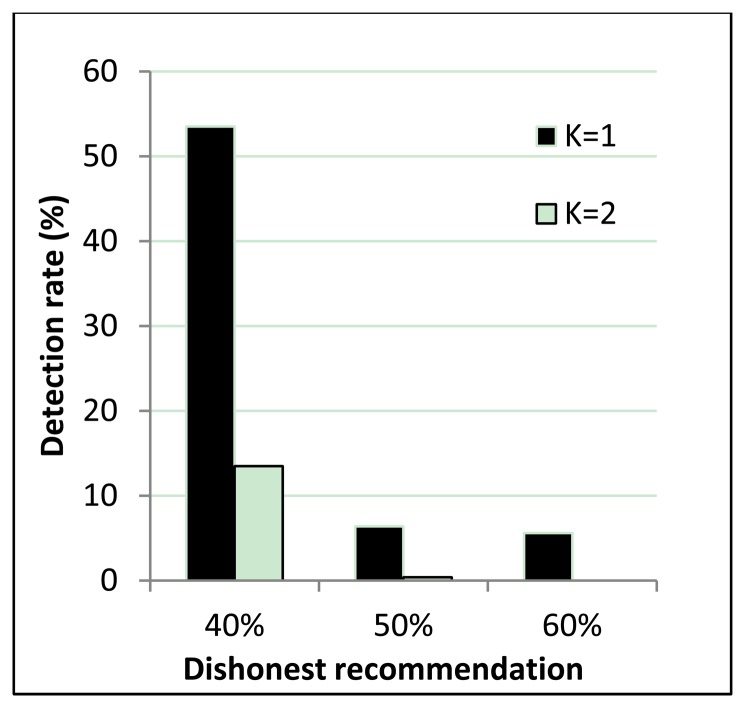
Dishonest recommendation detection.

However, the resilience of the one-step M-estimator is degraded when the percentage of dishonest recommendations increases to 50% to 60%. Results in Figure 8 are correlated with Figure 9, which shows that when the percentage of dishonest recommendations increases to 50% and 60%, dishonest recommendation detection becomes less than 10%. Evaluation results from Figures 8 and 9 show that a more suitable outlier threshold is K = 1. Moreover, recommendations can be securely aggregated when dishonest recommendations constitute up to 40% of the total recommendations.

## 5. Analysis of the Upper and Lower Bounds of Estimated Trust Values in Persistent Malicious Behavior

In this section, we show the upper and lower bounds of estimated trust values in persistent malicious behavior.

**Definition:** Node *x* is said to be malicious continuously when measured misbehavior is larger than zero, *a_x_*>0, all the time.

Hence according to our trust estimation model, estimated trust values will be as follows:

(15) $$\begin{array}{c} T_{x}(t)>T_{x}(t+\Delta )>T_{x}(t+2\Delta )\ldots \ldots .\geq T_{x}(t+n\Delta )\\ \Rightarrow T_{x}(t)>\frac{T_{x}(t)1-a_{x}(t+\Delta )}{2+M_{x}(t+\Delta )}>\frac{T_{x}(t+\Delta )+1-a_{x}(t+2\Delta )}{2+M_{x}(t+2\Delta )}>\\ \ldots .\geq \frac{T_{x}(t+(n-1)\times \Delta )+1-a_{x}(t+n\Delta )}{2+M_{x}(t+n\Delta )} \end{array}$$

For the sake of simplicity, we assume that forget factor(*S*) and measured misbehavior are fixed values. Moreover, trust value at time *t* equals one, *T_x_*(*t*) = 1. If *T_x_*(*t*) = 1 then aggregated misbehavior at time *t* will be zero, *M_x_ (t)*=0.

**Lemma 1:** *a_x_* ≤ *M_x_*(*t*+*n*Δ)≤1, for *n* ≥ 1

**Proof:**

*M_x_*(*t*+*n*Δ) = min((*a_x_*+(1−*S*))*M_x_(*t*+(*n* − 1)*Δ)),1) ≤ 1
*M_x_*(*t*+Δ) = *a_x_* as *Tx*(*t*) = 1

Assume that *M_x_*(*t*+*n*Δ)≥*a_x_*. Then since 1−*S*≥0 and *M_x_*(*t*+(*n*−1)*Δ)≥0, we have:

*a_x_*(*t*+(*n*+1)*Δ)+(1−*S*)*M_x_(*t*+n*Δ)≥*a_x_*.

Because 1≥*a_x_*. We have:

*M_x_*(*t*+(*n*+1)*Δ) ≥ min((*a_x_*(*t*+(*n*+1)*Δ)+(1−*S*)*M_x_(*t*+n*Δ),1) ≥ *a_x_*.

Next, we define two sequences, *c_n_* and *b_n_*, as follows:

(16) $$(\begin{array}{l} c_{1}=T_{x}(t)=1,\\ c_{n}=\frac{c_{n-1}+1-a_{x}}{3}, \end{array}n\geq 2,$$

(17) $$(\begin{array}{l} b_{1}=T_{x}(t)=1,\\ b_{n}=\frac{b_{n-1}+1-a_{x}}{2+a_{x}}, \end{array}n\geq 2.$$

Then, we can show that *c_n_* and *b_n_* become the lower and upper bounds of *T_x_*(*t*+(*n*−1)*Δ) given as follows:

(18) $$\begin{array}{ll} c_{n}\leq T_{x}(t+(n-1)*\Delta )\leq b_{n} & \left(\text{n}\geq 1\right) \end{array}$$

More detailed derivation is given in the following proposition.

**Proposition 1:** *c_n_* ≤ *T_x_*(*t*+(*n*−1)*Δ) ≤ *b_n_* (n≥1)

**Proof:**

First we consider the case where n = 1. Since *T_x_*(*t*+0*Δ) = *T_x_*(*t*) = 1, we can obtain the following relations from Equations (16) and (17):

$$\begin{array}{l} c_{1}=1,\\ b_{1}=1. \end{array}$$

Thus, we have *c*_1_=*T_x_*(*t*)=*b*_1_.

We assume that following relations are valid for *n* = *k* (*k* ≥ 1):

(19) $$\begin{array}{l} T_{x}(t+(k-1)*\Delta )\geq c_{k},\\ T_{x}(t+(k-1)*\Delta )\leq b_{k}. \end{array}$$

*T_x_*(*t*+*k*Δ) can be expressed as $T_{x}(t+k*\Delta )=\frac{T_{x}(t+(k-1)*\Delta )+1-a_{x}}{2+M_{x}(t+k*\Delta )}$.

Since *a_x_* ≤ *M_x_*(*t*+*n*Δ) ≤ 1 for *n* ≥ 1, by Lemma 1, we can obtain:

$$\frac{T_{x}(t+(k-1)*\Delta )+1-a_{x}}{3}\leq T_{x}(t+k*\Delta )=\frac{T_{x}(t+(k-1)*\Delta )+1-a_{x}}{2+M_{x}(t+k\Delta )}\leq \frac{T_{x}(t+(k-1)*\Delta )+1-a_{x}}{2+a_{x}}.$$

Combining the above relation and Equation (19) yields $\frac{c_{k}+1-a_{x}}{3}\leq T_{x}(t+k\Delta )\leq \frac{b_{k}+1-a_{x}}{2+a_{x}}$.

Combining Equations (16) and (17) and the above relation yields *c_k_*_+1_ ≤*T_x_*(*t*+*k*Δ)≤*b_k_*_+1_.

Thus, the proof is done by induction.

In more detail *c_n_* and *b_n_* can be expressed as follows (a detailed derivation is given in the Appendix):

(20) $$\begin{array}{l} c_{n}=\frac{1-a_{x}}{2}+{\left(\frac{1}{3}\right)}^{n-1}*\left(\frac{1+a_{x}}{2}\right),\\ b_{n}=\frac{1-a_{x}}{1+a_{x}}+{\left(\frac{1}{2+a_{x}}\right)}^{n-1}*\left(\frac{2a_{x}}{1+a_{x}}\right). \end{array}$$

Combining Equations (18) and (20) yields:

$$\begin{array}{l} \frac{1-a_{x}}{2}+{\left(\frac{1}{3}\right)}^{n-1}*\left(\frac{1+a_{x}}{2}\right)\leq T_{x}(t+(n-1)*\Delta )\leq \frac{1-a_{x}}{1+a_{x}}+{\left(\frac{1}{2+a_{x}}\right)}^{n-1}*\left(\frac{2a_{x}}{1+a_{x}}\right)\\ \Rightarrow \frac{1-a_{x}}{2}\leq \underset{n\to \infty }{\lim}T_{x}(t+(n-1)*\Delta )\leq \frac{1-a_{x}}{1+a_{x}}. \end{array}$$

From Equation (20), we find that the lower bound *c_n_* approaches the upper bound *b_n_* as *a_x_* approaches 1. Since the lower bound *c_n_* and the upper bound *b_n_* decreases with respect to *n*, we assume that *T_x_ (t*+*(n*-1)*Δ*)* has the same decreasing trend of Equation (15) in general. The smaller the gap between the upper and lower bound, the more similar decreasing trend of *T_x_ (t*+*(n*-1)*Δ*)* will be. As *a_x_* approaches to one, gap between the lower bound and upper bound decreases accordingly. So, in this case, decreasing trend of *T_x_ (t*+*(n*-1)*Δ*)* will be same with decreasing trend of the upper and lower bound.

## 6. Conclusions

This paper proposes a novel trust establishment scheme, which enables us to detect persistent malicious behavior and improves detection of on-off attacks. Moreover, it proposes using a one-step M-estimator, which helps aggregate recommendations securely. To the best of our knowledge, this is the first persistent malicious behavior detection enabled trust mechanism. The novelty of the scheme arises from comprehensively considering history and current status of the node and combining them intelligently. Evaluation results and theoretical analyses prove that it allows detection of consistent malicious behavior and on-off attacks. Moreover, recommendations can be securely aggregated using the proposed scheme when the percentage of dishonest recommendations is up to 40%. As a future work, implementation of the proposed trust scheme in *Ad hoc* On-Demand Distance Vector Routing (AODV) is being designed to estimate the performance of algorithm. Moreover, analyses on overhead of trust establishment in terms of resource consumption such as energy, memory, and computation are being considered as nodes are resource constraint in wireless sensor networks (WSNs).

## Figures and Tables

**Table 1.** Parameters to emulate persistent misbehavior.

**Parameters**	**Value**
Measured misbehavior	Fixed from 0.1 to 0.4
	Random between 0.1 and 0.4
Forgetting factor (*S*)	0.6
Trust estimation time interval	Δ
Trust threshold (*Q*)	0.6 and 0.5 (60 and 50 for GTMS)
Experiment time	50 Δ
Initial trust value	1

**Table 2.** Parameters to emulate an on-off attack.

**Parameters**	**Value**	

Duration of the on period	Randomly generated between [1;5] Δ	
Duration of the off period	Randomly generated between [1;5] Δ	
Number of instances of good behavior	On period	Randomly generated between [5;10]
	Off period	Randomly generated between [1;10]
Number of instances of bad behavior	On period	Randomly generated between [1;5]
	Off period	0
Forgetting factor	0.6 and 0.7(60 and 70 for GTMS)	
Trust estimation time interval	Δ	
Experiment time	75 Δ	
Initial trust value	1	
Trust threshold	0.6 and 0.7	

**Table 3.** Parameters to emulate bad mouthing attack.

**Parameters**	**Value**

Number of recommendations in each aggregation	10
Value of sincere recommendations	Randomly generated between [0.6;0.9]
Value of dishonest recommendations	Randomly generated between [0.3;0.5]
Trust threshold(*S*)	0.6 and 0.5 (60 and 50 for GTMS)
Number of aggregation experiments	50
Outlier detection threshold	K = 1 and K = 2

## Appendix

### Derivation of *b_n_* and *c_n_*

(i) To resolve *c_n_* from the recursive relation of Equation (16), we first determine *α* of the following relation:
  $$c_{n}-\alpha =\frac{1}{3}(c_{n-1}-\alpha )$$
  Since $c_{n}=\frac{1}{3}c_{n-1}+\frac{2}{3}\alpha =\frac{1}{3}c_{n-1}+\frac{1}{3}(1-a_{x})$, comparing this relation with equation(14) yields $\alpha =\frac{1-a_{x}}{2}$.
  Then, we can obtain
  $$\begin{array}{l} c_{n}-\frac{1-a_{x}}{2}=\frac{1}{3}(c_{n-1}-\alpha )={\left(\frac{1}{3}\right)}^{n-1}\left(c_{1}-\frac{1-a_{x}}{2}\right)={\left(\frac{1}{3}\right)}^{n-1}\left(\frac{1+a_{x}}{2}\right)\\ \Rightarrow c_{n}=\frac{1-a_{x}}{2}+{\left(\frac{1}{3}\right)}^{n-1}\left(\frac{1+a_{x}}{2}\right). \end{array}$$
(ii) To resolve *b_n_* from the recursive relation of Equation (17), we first determine *β* of the following relation:
  $$b_{n}-\beta =\frac{1}{2+a_{x}}(b_{n-1}-\beta ).$$
  This relation can be changed into
  $$b_{n}=\frac{b_{n-1}}{2+a_{x}}+\left(1-\frac{1}{2+a_{x}}\right)\beta =\frac{b_{n-1}}{2+a_{x}}+\frac{1+a_{x}}{2+a_{x}}\beta .$$
  Comparing this relation with Equation (17) yields
  $$\beta =\frac{1-a_{x}}{1+a_{x}}.$$
  Then, we can obtain
  $$\begin{array}{l} b_{n}-\frac{1-a_{x}}{1+a_{x}}=\frac{1}{2+a_{x}}\left(b_{n-1}-\frac{1-a_{x}}{1+a_{x}}\right)={\left(\frac{1}{2+a_{x}}\right)}^{n-1}\left(b_{1}-\frac{1-a_{x}}{1+a_{x}}\right)={\left(\frac{1}{2+a_{x}}\right)}^{n-1}\left(\frac{2a_{x}}{1+a_{x}}\right)\\ \Rightarrow b_{n}=\frac{1-a_{x}}{1+a_{x}}+{\left(\frac{1}{2+a_{x}}\right)}^{n-1}\left(\frac{2a_{x}}{1+a_{x}}\right). \end{array}$$
